# High sensitivity troponins: A potential biomarkers of cardiovascular risk for primary prevention

**DOI:** 10.3389/fcvm.2022.1054959

**Published:** 2022-11-30

**Authors:** Luis Leite, Pedro Matos, Antonio Leon-Justel, Claudio Espírito-Santo, Luis Rodríguez-Padial, Fernando Rodrigues, Domingo Orozco, Josep Redon

**Affiliations:** ^1^Cardiology Department, Coimbra University Hospital, University of Coimbra, Coimbra, Portugal; ^2^APDP e Hospital CUF Infante Santo, Lisbon, Portugal; ^3^Department of Laboratory Medicine, Virgen Macarena University Hospital, Seville, Spain; ^4^Primary Health Unit Buarcos, Buarcos, Portugal; ^5^Servicio de Cardiología, Hospital Virgen de la Salud de Toledo, Toledo, Spain; ^6^Clinical Pathology, University of Coimbra, Coimbra, Portugal; ^7^Department of Clinical Medicine, Miguel Hernández University, Elche, Spain; ^8^INCLIVA Research Institute, University of Valencia, Valencia, Spain; ^9^CIBERObn Institute of Health Carlos III, Madrid, Spain

**Keywords:** biomarkers, high sensibility troponin, troponin I, troponin T, cardiovascular risk, primary prevention

## Abstract

There have been several approaches to building charts for CV risk, all of which have both strengths and limitations. Identifying early organ damage provides relevant information and should be included in risk charts, although the direct relationship with risk is imprecise, variability between operators at the time to assess, and low availability in some healthcare systems, limits its use. Biomarkers, like troponin (cTns) isoforms cTnI and cTnT, a cardiac specific myocyte injury marker, have the great advantage of being relatively reproducible, more readily accessible, and applicable to different populations. New and improved troponin assays have good analytical performance, can measure very low levels of circulating troponin, and have low intra individual variation, below 10 %. Several studies have analyzed the blood levels in healthy subjects and their predictive value for cardiovascular events in observational, prospective and *post-hoc* studies. All of them offered relevant information and shown that high sensitivity hs-cTnI has a place as an additional clinical marker to add to current charts, and it also reflects sex- and age-dependent differences. Although few more questions need to be answered before recommend cTnI for assessing CV risk in primary prevention, seems to be a potential strong marker to complement CV risk charts.

## Introduction

Global CV risk charts aim to answer key questions that direct stratification (1–3). There have been several approaches to building charts for CV risk, all of which have both strengths and limitations (4–14). The strengths of the global CV risk scores depend on the quality of the original data and the methodology applied (3) for internal and external validation, as well as calibration for specific populations. There are several limitations, including:

(i) Old prospective cohorts–where the calculation was made using data from the past, there is a bias which overestimates risk due to the decrease in the overall trend in disease incidence;
(ii) Age–while young people will have a very low estimated CV death risk, older men will have an estimated CV death risk exceeding 5–10 %, even when CV risk factors are low;
(iii) Applying risk-estimation scores to regions with different baseline rates of CV disease will lead to either under- or overestimation;
(iv) Low sample sizes in the original study cohort.

The implementation of these guidelines is a basic tool for CV risk prevention in family medicine (primary care). However, it is difficult to choose which risk prediction chart to use from all those currently available since the results can differ from one to another (15–17). Attempts have been made to improve the performance of score charts introducing modifiers (14) or machine learning (18, 19).

Identifying early organ damage provides relevant information and should be included in risk charts. The presence of early organ damage provides an estimation of the impact of CV risk factors, how long were they present and the individual risk due to genetic predisposition or other factors. Calcium score, presence of carotid plaque, left ventricular hypertrophy, carotid-intima media thickness, urinary albumin excretion and glomerular filtration rate have all been used in this context. However, direct relationship can be imprecise for some of these markers, like carotid plaque or left ventricular hypertrophy, as there is sometimes variability between operators and also low availability for others, like calcium score, in some healthcare systems may limit its use.

Biomarkers have the great advantage of being relatively reproducible, more readily accessible, and applicable to different populations (20, 21). The role of certain cardiac markers in diagnosing acute myocardial infarction is well established, Troponin (cTns) is included in the definition of the disease (22), likewise, natriuretic peptides (cNPs) included for the diagnosis of heart failure (23). A vast number of studies indicate that both cTns and cNPs are able to detect the individuals at higher cardiovascular risk in the general population. The measurement of cTns and cNPs gives different, but complementary, pathophysiological and clinical information. A contemporaneous increase of the two cardio-specific biomarkers suggests that some powerful stressor mechanisms have already caused relevant alterations on both cardiac function and cellular structure (24).

Recently, it has been suggested that the contribution of specific cardiac biomarkers in risk assessment provides useful information but needs to fulfill a set of criteria. Relevant biomarkers need to:

i) Be specific for cardiac damage;
ii) Predict future CV events and mortality in the general population;
iii) Be responsive, with reduced levels following intervention and treatment;
iv) Change in parallel with a reduction in risk;
v) Confer a low-cost per quality-adjusted life years gained.

## CV biomarkers

There are several studies that have investigated CV biomarkers as potential tools for assessing risk. Some of those most studied include biomarkers of myocardial stretch (natriuretic peptides), inflammation (newly emerging biomarkers), and myocyte injury (troponin). A biomarker, whether in isolation or in combination with other measurable factors, can be applied to stratification charts, and has a potential role in calculating restratifying risk, reaching early diagnosis, selecting better therapy approaches, and potentially preventing worse outcomes for patients. However, it is essential that there is a clear understanding of the pathophysiology of each biomarker, as well as its analytical performance, and which factors influence its variability.

The most recent European Heart Association guidelines suggest some novel uses for cNP in HF with ejection fractions of 40–50 % and above 50 %, however, more data is needed to understand what the cut-off should be for this biomarker before introducing it into the risk charts, although based in the data of the STOP-HF trial American Guidelines recommended that BNP can be used as a screening tool for heart failure risk (25). The specificity of cNPs as cardiac biomarkers is in question because they can also be raised in other non-cardiac conditions, such as obesity. There are some notable clinical examples where NPs appear particularly useful as reference biomarkers:

(i) In patients presenting with symptoms of cardiac amyloidosis;
(ii) For predicting cardiotoxicity in patients undergoing chemotherapy;
(iii) In the early detection of CV events in patients with systemic inflammation, such as systemic lupus erythematous, and neuromuscular diseases (26).

Among inflammatory biomarkers, C-reactive protein (CRP) has been used to estimate the inflammatory component of CV risk and hs-CRP is used by American guidelines as a risk enhancer, but its use in risk stratification has been challenged. Two emerging biomarkers for inflammation and fibrosis, particularly for HF, are galectin 3 (Gal-3) and soluble ST2 receptor (sST2r)–a member of the interleukin-1 receptor family (27, 28). Gal-3 is involved in both the acute and chronic responses to inflammation and is known to promote tissue fibrogenesis. It may play a role in the cardiac remodeling process by stimulating collagen production and deposition by myofibroblasts in response to acute damage, either ischemic or non-ischemic. However, it is not cardiac specific as levels can also be elevated during infection or in chronic kidney disease. Some studies suggest sST2r is a possible biomarker for prognosis in HF patients, especially as it is not influenced by kidney function, but it cannot be used diagnostically because it can also be elevated in several inflammatory conditions, and in chronic obstructive pulmonary disease (29).

Cardiac troponins (cTns) cTnI and cTnT have been the most successful cardiac-specific circulating biomarker in cardiovascular (CV) medicine. Why? Because they have: (1) improved dramatically the differential diagnosis of acute chest pain, providing a reliable means for the accurate diagnosis of acute coronary syndromes (22, 23). (2) Shown pathological elevation in observed in many disease states and considered an independent prognostic marker in several CV and non-CV conditions, such as ACS, chronic coronary artery disease, acute and chronic heart failure, cancer therapy-related cardiotoxicity, and chronic kidney disease (26).

Said that, one of the key analytical requirements of a cTns assay today, in order to be characterized as “high-sensitivity,” is its detectability in >50% of apparently healthy individuals, as set by the International Federation of Clinical Chemistry and Laboratory Medicine (30). This considerable detectability in asymptomatic individuals gave rise to the hypothesis that hs-cTn could be used for the stratification of CV risk in the general population.

## Troponins

Cardiac troponin is a cardiac specific biomarker linked to cardiac outcomes (see below) as well as cardiac imaging (31). High sensitivity troponin levels, hs-cTn, have been correlated to coronary calcium scores (32) and the development of unrecognized myocardial infarctions detected by magnetic resonance image (33) in individuals over 70 years old with no CVD. Several studies have shown that high sensitivity hs-cTnI has a place as an additional clinical marker to add to current charts, and it also reflects sex- and age-dependent differences (34).

### Biological meaning

Troponin is a contractile protein which can be found in three isoforms: C (cTnC), I (cTnI) and T (cTnT), with sizes 18, 23, and 35 kD respectively. They are all involved in the contraction and relaxation of the myocardium. cTnC is responsible for the Ca^2+^ binding subunit, cTnI inhibits actin-myosin contraction and shuttles between binding actin and cTnC in response to intracellular calcium concentration, and finally cTnT is responsible for binding troponin to tropomyosin (35).

Studies show that the release of troponin into the blood stream can be a consequence of reversible injury; it is associated with normal myocyte turnover, the cellular release of proteolytic degradation, an increase of wall permeability, or membranous blebs. On the other hand, the mechanisms of irreversible injury can be associated with tissue necrosis, due to hypoxia and apoptosis. Once in the blood stream, different enzymes are responsible for their degradation into smaller products, which range from 12 to 23 kD. This degradation mainly occurs through proteolysis where calpains, caspases, cathepsin L and gelatinase A play important roles. Some degradation is also caused by transglutamination. Finally, these products are cleared from the body, either by endocytosis in the reticuloendothelial system of the spleen, liver and bone marrow, or excreted through the renal system (36).

Although all three isoforms can be found in the heart, cTnI and cTnT are cardiac specific. This feature is key to making troponin measurement not only the gold standard method for ruling-in or ruling-out patients with suspected acute coronary syndrome in the Emergency Department, but also to classify myocardial infarction into five levels according to the Fourth Universal Infarction Consensus Document published in 2018 (37). Interestingly enough, the TROPIC study concluded that peak levels of both troponin assays predicts events at 30 days, but cTnI may be more accurate than cTnT in this population of one million patients. In addition, a published review in CKD patients with and without dialysis, in absence of acute coronary syndrome (38), elevated of cTnT and cTnI, were associated with worse prognosis.

One of the pressing issues to address is understanding the reason behind low levels of troponin in the circulation of asymptomatic individuals. This could be due to myocardial renewal/remodeling (39), pre-load mechanical stretch (40), the activation of the adrenergic and renin-angiotensin-aldosterone systems (41), and a sign of subclinical coronary atherosclerosis (42).

Recently, the genetic contribution to cardiac TnI concentrations and its causal effect on CV phenotypes has been analyzed in a genome-wide association study (GWAS) with Mendelian randomization analysis on subjects enrolled in the Trøndelag Health Study and the Generation Scotland Scottish Family Health Study (43). The results support hs-cTnI as a non-causal marker of acute myocardial infarction and heart failure.

### Methods of measurement

With increasing numbers of the population being tested for new biomarkers and the need to measure them at very low circulating levels, understanding the analytical performance of the assays themselves is becoming critical for both laboratory staff and clinicians.

High sensitivity assays require gold standard analytical performance. For an assay to be defined as having high sensitivity (44), two analytical criteria must be met. The first is that there should be an optimal total imprecision or coefficient of variability (CV) below 10% at the 99^th^ percentile upper reference limit (URL) in acute settings. Secondly, the concentration of the biomarker needs to be above the limit of detection (LoD) for over 50 % of individuals who have no evidence of CVD. The low precision of some tests has an obvious impact on clinical decisions, and some studies have shown that there is a 10 % misclassification of patients due to assay bias and imprecision, particularly at the low concentration thresholds. The mean prevalence of detectable cTnT and cTnI in the general population is currently around 58 % (ranging from 25 to 66.5%) and 78% (ranging from 74.8 to 93%), respectively, and is typically lower in the younger population (31).

New and improved troponin assays have good analytical performance, can measure very low levels of circulating troponin, and have low intra individual variation (below 10 %), allowing clinicians to easily follow changes in patients over time. Currently, there are only two assays available that have received the CE mark for risk stratification in the general population (45).

At the time of clinical presentation, there are a number of analytical interferences that need to be considered (46). They include:

(i) *A* high degree of ***hemolysis*** can occur if a sample waits around too long before testing, and this directly correlates with an increased rate of false negative results, potentially leading to a misclassification of risk.
(ii) ***Biotin supplements*** are frequently found in hair and beauty treatments, and are recommended for alleviating symptoms in multiple sclerosis, but they can yield false positive results.
(iii) ***Antibody interference*** can lead to false positive or false negative assay results.
(iv) Patients with ***injured skeletal muscle such as that found in myopathies***, since the skeletal isoform of troponin is detected by some assays instead of the cardiac form. Overall, cTnT is more prone to these assay interferences than cTnI, with higher variability in assay results.

There are also some other instances where raised levels of cTnI may not necessarily be related to a heightened risk of heart disease. Some healthy individuals, for example, have a degree of circulating troponin that relates to the normal physiological turnover of cardiomyocytes. It is also important to bear in mind that troponin levels will increase with age and differ according to sex, so cut-offs will require adjustment according to these parameters (47). Moreover, renal function needs to be taken into account since low glomerular filtration rate decreases troponin clearance (48, 49).

### Troponin levels in healthy subjects

Several studies have reported the prevalence of low levels of troponins in healthy subjects. In a small cohort, troponin as measured on the ARCHITECT STAT Troponin I was detectable in 96% of cases, with a LoD of 1.9 and a 26 ng/L value for the 99^th^ percentile (50). In the Generation Scotland Scottish Family Health Study of nearly 20,000 subjects, cTnI was detected in 74.8% and cTnT in 53% (51). A meta-analysis of data from 28 long-term prospective studies, covering a total of 154,052 participants with no CV disease (52), showed that circulating cTnI and cTnT are detectable in 80% of healthy subjects below the 99^th^ percentile URL. The data from the BiomarCaRE consortium, looking at 74,738 participants, suggest that reference values should be different for men and women (53). Likewise, in the Generation Scotland Scottish Family Health Study, levels of cTnI and cTnT were lower in women compared to those in men but they were greater prognostic markers. In this study, 19,501 individuals were analyzed measuring cTnT and cTnI. CV risk factors were associated with both troponins but cTnI was more strongly associated with age, male sex, body mass index and systolic blood pressure as compared to cTnT, while cTnT was more associated to diabetes (54). A registry in India, the Apollo Abbott Cardiac Registry, is currently using a risk registry approach to categorize patients according to risk prediction calculators and high sensitivity troponin I. The follow-up of these patients is ongoing to evaluate and understand changes in risk over time and discover how this can guide therapy selection.

### Predictive value of troponin levels in healthy subjects

Several observational, *post hoc* analyses of previous trials and follow-up studies have been used to assess the potential value of troponins, both cTnI and cTnT, as relevant markers of risk in healthy people.

#### Observational

##### cTnI

- In a Scottish Heart Health Extended cohort of 15,340 subjects followed over 20 years, cTnI is an independent predictor of CV events (55).
- In the BiomarCaRE study, hs-cTnI was associated independently with the long-term risk of CV mortality, morbidity, and all-cause mortality. The risk increases across the quintiles of troponin I levels. Elevated cTnI concentrations are associated with an increased risk of the incidence of stroke in the community, irrespective of the subtype. Adding cTnI concentrations to classical risk factors only modestly improved the estimation of the 10-year risk of stroke in the overall cohort, but might be of some value in individuals at an intermediate risk (56).
- In the prospective Akerhus Cardiac Examination 1950 Study, the concentration of cTnI is predictive of carotid atherosclerotic burden, assessed by a carotid plaque score and intima-media thickness (42).
- Genetically predicted elevated circulating cTnI concentrations were associated with a higher risk of atrial fibrillation and cardioembolic stroke (57).
- Elevated cTnI in older women with abdominal aortic calcification were associated with a higher risk of atherosclerotic vascular disease mortality and all-cause mortality (58).
- Elevated cTnI indicated a higher risk of coronary artery disease in non-alcoholic fatty liver disease (59).

##### cTnT

- In subjects with an absence of CV events from the Dallas Heart Study, cTnT levels were associated with an incremental risk (60, 61).
- In the Atherosclerosis Risk in Communities (ARIC) study, cTnT levels in the highest category of >14 ng/L significantly increased the risk of CHD, fatal coronary events, total mortality and heart failure. Even minimally elevated levels were associated with risk of heart failure and mortality (62).
- In the Cardiovascular Health Study of community-dwelling adults aged 65 years or older, increments of >50% of cTnT were associated with an increased risk of heart failure and mortality (63).
- In the Women Heart Study, cTnT levels were associated with total CV disease and CV mortality in diabetics, but not in non-diabetics (64), and with heart failure in early menopausal women (65).
- In a Chinese population, cTnT levels predicted risk for major CV events and all-cause mortality (66).
- In the TUSARC Registry, elevated cTnT was significantly associated with the incidence of major CV combined end points, heart failure, and CV and all-cause mortality in asymptomatic individuals at very high CV risk (67).
- In a study with a short follow-up time of 796 days, cTnT provided a superior prognostic value to the ESH-SCORE for the prediction of all-cause mortality and a composite end point in stable outpatients, with and without relevant CV disease (68).
- The ARIC study of an elderly population of >66 years old, with a median follow-up of 6.3 years, showed that cTnI improves mortality and CVD risk stratification in older adults beyond traditional risk factors, and improves model discrimination better than cTnT for certain outcomes, CV mortality and heart failure (69).
- Finally, is worthy to mention the prognostic value of both troponins in the risk to develop heart failure in data from 4 community-based longitudinal cohorts with adjudicated heart failure endpoints: the FHS (FraminghamHeart Study), the PREVEND (Prevention of REnal and Vascular Endstage Disease), the MESA (Multi-Ethnic Study of Atherosclerosis), and the CHS (Cardiovascular Health Study) (70). The study concluded that troponins shown strongly increment of risk for incident heart failure in both sexes although improvement of risk prediction above and beyond an established clinical HF model is limited.

#### Post-hoc analysis

##### cTnI

- The Dallas Heart study observed that cTnT values higher than the 99^th^ percentile in a multiracial population increased the adjusted risk of all cause mortality 2.8-fold, adjusted by traditional risk factors (60).
- The ARIC study also confirms that troponin I was more closely associated with the incidence of CHD in women than it was in men (71). More recently, another trial showed that hs-cTnI improved mortality and CVD risk stratification in older adults beyond traditional risk factors, and improved model discrimination more than cTnT for certain outcomes. Elevated cTnI without CVD identifies a high risk group with a comparable mortality risk to those with a history of clinical CVD (69).
- In the JUPITER study, cTnI concentrations in the highest tertile were significantly associated with a first major CV event, and with a higher all-cause mortality. The benefits of rosuvastatin were consistent regardless of the baseline cTnI (72).
- Analysis from the Further Cardiovascular Outcomes Research With PCSK9 Inhibition in Subjects With Elevated Risk (FOURIER) identify that plasma cTnI levels predicted increased CV risk in both very-high and no very high risk. Furthermore, in the last group plasma levels of cTnI >6 ng/L identified 25% of patients with a similar event risk that those classified as very high risk. Not only this, the benefit of evolocumab treatment was equal that the obtained in the very high risk (73).

##### cTnT

- The SPRINT study, designed to assess the correlation of a reduction in CV risk with a drastic drop in blood pressure, identified that patients with the highest hs-cTnT values are associated with a higher mortality, and that blood pressure lowering treatment reduces most of the risk in these patients (74).
- In patients in the Chronic Renal Insufficiency Cohort (CRIC), the fourth quartile of cTnT levels increased the risk in stroke, compared to the first quartile (75).
- In patients with a cohort of Type II diabetes mellitus followed by 11 years, the patients with undetectable hs-cTnT levels (<3 ng/L), only 23% died, compared with 58% with low detectable levels (3–14 ng/L) and 84% with raised levels (≥14 ng/L) (76).

#### Meta-analysis

##### cTnI

- Pooled estimates in the Willeit meta-analysis (50) of 28 long-term prospective studies suggested that individuals with cardiac troponin values in the top third of the population distribution are at a 43% increased risk of any CVD, a 59 % increased risk of CHD, and a 67% increased risk of fatal CVD outcomes. A smaller but significant increase in stroke risk, 35%, was also noted.

#### Prospective study

##### cTnI

- The 2016 WOSCOPS (77, 78) study showed that a baseline troponin was an independent predictor of myocardial infarction or death from CHD. Additionally, a five-fold greater reduction in CV events was registered when troponin concentrations decreased by over a quarter, and treatment with pravastatin reduced the levels of troponin by 13%, consequently leading to better patient outcomes. This study also published a guideline illustration of how to apply troponin for risk stratification purposes and for guiding and monitoring disease progression.
- The Trøndelag Health (HUNT study) (79) recruited around 9,000 subjects free of CV disease at a baseline and followed them for 13.9 years. cTnI of >10 ng/L in women and >12 ng/L in men was associated with a hazard ratio of 3.6, significantly higher that the obtained with 3 mg/L of hs-CRP in the risk of myocardial infarction, heart failure and cardiovascular mortality (80).

##### cTnT

- Serial measurement of cTnT in the Cardiovascular Health Study was significantly associated with the incidence of heart failure (81).
- Serial measurement of cTnT over 6 years was significantly associated with the incidence of atrial fibrillation (82).

These findings suggest that it may be useful to integrate troponin into the established score charts, in conjunction with other factors. In the study of Blankenberg et al. (51), the addition of troponin I to the SCORE improved the prediction of CV disease, and of global and CV mortality risk, with the improvement being particularly important above the age of 65. The incremental prognostic value of cTns related to its cardiac specificity, may help to address the residual CV risk not covered by established prognostic markers and tools. However, further investigation is needed in order to apply this strategy to the correct population groups.

### Potential use of the hs-cTn bioassay in risk assessment

Current score charts are clearly insufficient as they stand. Prospective studies will be a very useful tool to have in future to investigate risk in primary prevention, despite their long follow-up times. Integrating tools such as cTnI, but also including others such as calcium score level, is a strategy that warrants further discussion and a stratification plan of action moving forward.

There are two scenarios to explore. In primary prevention, there is a need to evaluate individual risk scores and an extra tool will be necessary, either calcium score or cTnI, or both. The other is secondary prevention, where troponin could help in better defining real CV risk and therapeutic decision making. Defining which patients have a higher real thrombotic risk through the measurement of troponin may be of real benefit, namely with more aggressive therapeutic strategies and eventual combination hypocoagulation/antiaggregation therapy.

### Algorithm for using troponin I as a marker of risk

Grounded information about how, when and which group of healthy subjects would benefit from using cTnI to refine CV risk is limited, however, one algorithm has been proposed as a reasonable approach (83). The algorithm proposes using cTnI in subjects with moderate risk of between 1 and 5 % according to SCORE. In this group, subjects with cTnI levels of 6 and 4 ng/L in men and women, respectively, would be given lifestyle advice, those with 6 to 12 and 4–10 ng/L, respectively, would be recommended to make aggressive lifestyle changes, and those with >12 or 10 ng/L would receive similar aggressive lifestyle interventions plus drug therapy for the CV risk factor present. Table 1, describe a proposal for recommendations in the use of cTnI based on evidence available up to now.

**Table 1:** Recommendations for the use of cTnI in primary and secondary prevention: A tentative approach.

**Score risk (%)**	**cTnI values (men/women)**	**Recommendation**
**Secondary prevention**		
Stablish ASCVD	>12 ng/L men >10 ng/L women	High CV risk. CV risk factors Treatment. Intensify pharmacological treatment
**Primary prevention**		
>5–10%	>12 ng/L men >10 ng/L women	Check for possible cardiac injury. CV risk factors Treatment. Intense lifestyle advice plus treat underlying causes of risk is recommended.
1–5%	>12 ng/L men >10 ng/L women	Intense lifestyle advice plus CV risk factors treatment. Follow-up at 3 Month
	6–12 ng/L in men 4–10 ng/L in women	Follow up 6 months and Intense lifestyle advice. Follow-up at 6 Month
	6 ng/L in men 4 ng/L in women	Regular counseling: lifestyle advice if all other risk factors also low. Follow-up at as needed.

### Health economics and outcomes research

The value of health economics is to provide information on healthcare decisions, including exploring new opportunities, establishing new or replacing existing services or procedures, and deciding on funding and the allocation of resources. The purpose of these evaluations is to assess and compare different choices to inform the decision-making process, and many factors can influence them. Diagnostics in primary care is very complex because of population heterogeneity, the low incidence of a specific disease, and the variation in treatment and management. A health economics study can be confirmatory or exploratory in nature. In the latter, it can help to inform the design of a clinical trial to test the impact of specific factors. In the cardiology field, systematic reviews of economic evaluations found that evidence regarding cost-effectiveness in primary prevention was inconclusive, as any modification or variation could influence the overall results (84).

A few studies have reported a clear association between troponin and long-term CV outcomes. However, some questions remain unanswered and health economics modeling is the only way to link the existing evidence and assess impact.

One study aimed to get an early estimation of the cost-effectiveness of troponin I using a screen and prevent strategy in two different countries–one with low risk, one with high–where patients in the high risk category based on troponin were treated with statins. This was compared to a do-nothing approach. The simulation calculated the number of CV events and deaths, the healthy and quality-adjusted life years, and the direct and indirect costs, over a 10-year period. The indirect costs were related to losses in productivity due to disease in a working population age (85, 86). Hazard ratios were derived from the Kaplan-Meyer curves in the HUNT study and, using a cohort of 25,000 individuals stratified according to low, moderate, and high risk based on the levels of sex-specific troponin, there was a significant decrease in CV events in the high risk group, down to 15%. In summary, the number of CV events per 1,000 individuals was reduced by five with the screen and prevent approach; 200 people would need to be screened to prevent one event from occurring. This corresponded to a risk reduction of 9 % in the general population, and of 43% in the high risk group. The results were similar for the two countries included in the study–Germany (low risk) and Kazakhstan (high risk)–and the strategy was found to be cost-effective in the first case and cost-saving in the latter. In terms of economic impact, the investment required for screening and prevention in both settings was offset by savings from lower medical costs–from fewer CV events–and a significant reduction in indirect costs caused by productivity losses.

### Summary and conclusion

Clinical trial data published in the last 5 years is showing good correlations between cTns levels, both cTnI and cTnT, and the risk of developing a future CV event. Other studies are ongoing that are evaluating cTnI as a predictive biomarker that responds to therapy and changes with risk modification. The mechanism by which a rise in cTnI levels correlates to a higher incidence of CV events is still unknown, and further understanding of its link to heart morphology and function is critical.

A few more questions need to be answered before a wider recommendation of using cTnI in CV prevention can be made. What patient groups can benefit more from cTnI measurements as an additional stratification in primary prevention? How can cTnI be used in secondary prevention strategies? And is cTnI useful for guiding therapeutic decisions and monitoring disease progression? In addition, further studies need to show its association with other CV risk factors and subclinical disease.

In the meantime, using cTnI assays for CV risk effectiveness will be influenced by several factors, such as which individuals are tested, which practices and score charts are used, whether troponin is used separately or in addition to the charts, and how the information is subsequently used to manage patients. Other factors that may influence the final costs are the implementation of the new biomarker, the uptake, and whether health check programs are already in place. The cost-effectiveness of these preventive strategies needs to be taken into consideration when making decisions for future guidelines in cardiology.

## Author contributions

LL, CE-S, and JR contributed to conception and design of the study. FR and DO organized the database. FR and JR performed the statistical analysis. PM, AL-J, and JR wrote the first draft of the manuscript. LL, PM, AL-J, CE-S, and LR-P wrote sections of the manuscript. All authors contributed to manuscript revision, read, and approved the submitted version.

## Conflict of interest

The authors declare that the research was conducted in the absence of any commercial or financial relationships that could be construed as a potential conflict of interest.

## Publisher's note

All claims expressed in this article are solely those of the authors and do not necessarily represent those of their affiliated organizations, or those of the publisher, the editors and the reviewers. Any product that may be evaluated in this article, or claim that may be made by its manufacturer, is not guaranteed or endorsed by the publisher.
